# Sharpening Precision Medicine by a Thorough Interrogation of Metabolic Individuality

**DOI:** 10.1016/j.csbj.2016.01.001

**Published:** 2016-01-21

**Authors:** Kirk Beebe, Adam D. Kennedy

**Affiliations:** Metabolon Inc., Durham, NC, USA

**Keywords:** Precision Medicine, Metabolomics, Inborn Errors of Metabolism, Mass Spectrometry, Genome Wide Association Study, Untargeted Analysis, Diagnostics

## Abstract

Precision medicine is an active component of medical practice today, but aspirations are to both broaden its reach to a greater diversity of individuals and improve its “precision” by enhancing the ability to define even more disease states in combination with associated treatments. Given complexity of human phenotypes, much work is required. In this review, we deconstruct this challenge at a high level to define what is needed to move closer toward these aspirations. In the context of the variables that influence the diverse array of phenotypes across human health and disease – genetics, epigenetics, environmental influences, and the microbiome – we detail the factors behind why an individual's biochemical (metabolite) composition is increasingly regarded as a key element to precisely defining phenotypes. Although an individual's biochemical (metabolite) composition is generally regarded, and frequently shown, to be a surrogate to the phenotypic state, we review how metabolites (and therefore an individual's metabolic profile) are also functionally related to the myriad of phenotypic influencers like genetics and the microbiota. We describe how using the technology to comprehensively measure an individual's biochemical profile – metabolomics – is integrative to defining individual phenotypes and how it is currently being deployed in efforts to continue to elaborate on human health and disease in large population studies. Finally, we summarize instances where metabolomics is being used to assess individual health in instances where signatures (i.e. biomarkers) have been defined.

## Highlights

• Untargeted biochemical profiling has the potential to phenotype individuals where genetic associations do not seem to show penetrance
• Metabolomics can be leveraged with other ‘omics data to discern phenotype information that is driven by environmental, microbiota, or epigenetic factors.
• Tracking the biochemical profile of individuals may help discern effectiveness or response to treatment or disease progression.

## 1 Introduction

Better health, improved diagnosis and treatment are aims that individuals and health care providers aspire to. As highlighted by President Barack Obama's Precision Medicine Initiative in 2014, precision medicine is at the heart of these aspirations. Precision medicine is essentially a combination of prevention and treatment strategies tailored for the individual. Although to do so in a widespread way at the individual level remains, in most cases, aspirational, examples and initiatives focusing at the individual level are becoming more prevalent [1], [2], [3], [4], [5], [6], [7].

In order to do this, one must be able to identify signatures (biomarkers) associated with diverse health and disease states and be able to react to these signatures with a tailored intervention. The foundation to being able to do this at the individual level is having a precise blueprint of human health. (By blueprint, we are referring to a detailed technical map of the molecular underpinnings of human health). A precise blueprint is required to support the identification of deviations from this blueprint and the design or implementation of modalities for applying corrections to these deviations. In order to have an expansive toolbox of precise recommendations about wellness and treatment for an individual, the collection and analysis of massive amounts of data are required.

### 1.1 A Blueprint Is Not That Simple

**Fig. 1:**
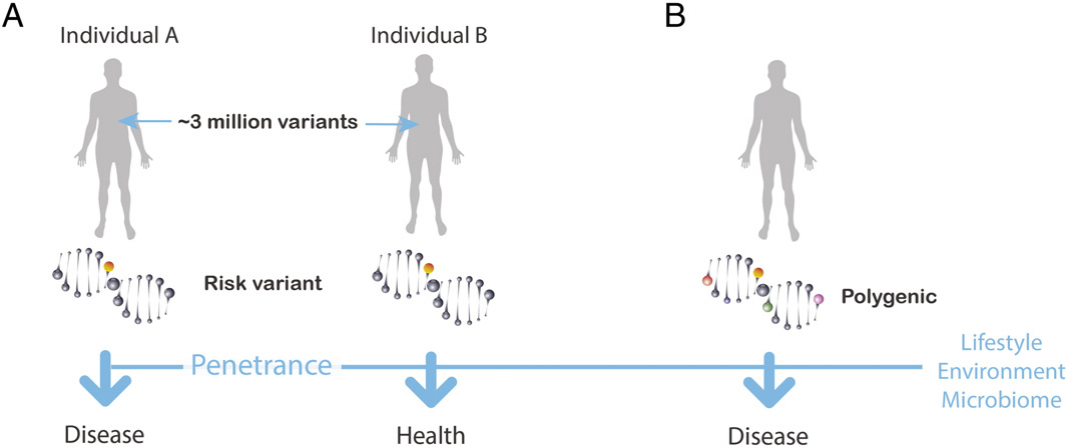
*Complex landscape for predicting allelic variants that impact health*. *A*. Single variant example. Even after delineating a variant that projects a high risk for developing a disease, outcomes can be very different where one individual may succumb to a deleterious variant while another individual with the same variant may remain healthy and never develop disease – the concept of penetrance. *B*. Multi/polygenic example. Many traits are influenced by a combination of allelic variants and therefore are more challenging to directly map to a trait. In both scenarios, external factors (lifestyle, environment, and the microbiome) frequently are involved in how the effects of these variants play out and whether an individual remains healthy or develops disease.

Deciphering the genome is one key for creating this blueprint. But, beginning with the first draft of the human genome and the subsequent years of genome research that followed, a striking degree of unanticipated (by many) complexity has emerged, making the hunt for genetic drivers of health and disease very challenging. Some of the more salient elements are elaborated below and in Fig. 1.

First, humans have strikingly high allelic variation [8], [9]. This high variation is likely a function of our genomes having an inherent adaptability embedded within them. A seminal example of this is the recent work illustrating genetically driven metabolic adaptation within Greenlandic Inuit populations who adapted to extreme weather and a lipid rich diet high in protein [10]. To adapt to a diet rich in omega-3 polyunsaturated fatty acids (PUFAs), this population was shown to bear specific alleles affecting enzymes that manage PUFAs (the fatty acid desaturases) which also have downstream effects on traits such as height. Presumably, some degree of plasticity in the human genome has conferred fitness for the diverse niches that humans have occupied across the planet. And, while this may be an extreme example, the statistics on allelic variation suggest that there is a range of adaptation throughout human populations. For example, it is estimated that any human genome would bear ~ 3 million variants compared to a reference genome [7], [11]. This high variation presents a tremendous challenge for deciphering the allelic variants that are important for health and disease from those that simply achieve statistically significant associations.

Even when a disease-causing genetic variant is identified, the likelihood of the individual bearing this variant getting the disease can frequently be modest to very low due to genetic penetrance [8], [9]. In contrast to genes that confer a nearly invariant risk of disease such as the gene for Huntington's disease (*HTT*), many genes are less penetrant or only reach high penetrance in the context of age such as BRCA1 (conferring ~ 50% penetrance) for breast and ovarian cancer and APOE-e1 for Alzheimer's disease. Many more genes attributed to disease risk have very low risk levels. Further, many confer risk within the context of environmental triggers such as the HLA-DQ2 gene and dietary gluten in the case of celiac disease. So, although high-risk single gene variants exist and more rare examples are certain to be discovered, the capacity of the body to compensate – through its complex networks – may make assigning absolute risk to a majority of mutations challenging.

Further, complicating the search for disease-causing genetic variants is the fact that most traits of interest are distributed across the genome – so-called multi- or polygenic traits [8], [9]. Thus, when sequencing an entire genome or exome, determining which variants that are key to a single trait have been challenging, particularly when the majority of variants identified so far impart relatively small increments of risk. Also, a majority of mutations of interest reside in non-coding regions [7] expanding the challenge of assigning function to an allele that associates with a given trait. There is a need to add data to help define of these non-coding regions (a function that has yet to be clearly defined [12].

All of the above points are not aimed to dissuade genome inquiry. Key insights to the blueprint required for precision medicine are embedded within the genome and many examples have already emerged with a range of suggested actionable mutations suggested to be as high as several hundred down to a more conservative – “unequivocal” – set of 57 genes recommended by the American College of Medical Genetics and Genomics (ACMG). These genes may indicate the presence of 24 disorders where intervention may reduce or prevent serious morbidity or early mortality. But, in order to achieve additional insights, sequencing (through whole genome or exome sequencing) of many more individuals will be required. Then, once sequenced, a massive data reduction challenge resides before consensus and actionable data can be effectively mined as illustrated by a recent whole genome sequencing (WGS) initiative published in JAMA where it was concluded that, while WGS will result in clinically actionable genetic variants, significant challenges remain in reducing the majority of this data to beneficial practice for individuals [11], [13].

Given the above challenges for mining the genome to decipher human health and disease, it is increasingly appreciated that orthogonal data streams will be required to help to identify the more relevant or “active” parts of the genome as well as to account for influences outside of genome sequences, particularly for complex diseases such as diabetes, cancer, cardiovascular and neurological diseases (the predominant causes of morbidity and mortality in the developed world), since most regard these diseases as being caused by a combination of genetic and environmental factors [14]. Influences like the microbiome [15] epigenetics and the possibility of transgenerational inheritance [16] continue to reveal their influence in human health and disease. These extra-genome influences make establishing this blueprint solely through mapping the genome extremely challenging.

### 1.2 What Is It Going to Take to Derive This Blueprint?

Clearly, the mapping of the genome is a cornerstone of this effort but having efficient ways to interrogate the data to identify alleles that have meaningful activity in influencing phenotypes is a challenge. Thus, having a means to detect the parts of the genome that “transmit-out” to actively influence the phenotype would be ideal. In essence, it is a way to detect the parts of the genome that are actively engaged in composing the phenotype. Then, once these alleles are identified, it would be ideal to have data that can inform on the function of the alleles.

Given the complexity of traits and the influence of factors such as epigenetics, and external factors such as the environment, lifestyle, and the microbiome, it also would be ideal to have a way to track the influence of these extra-genetic factors. Further, when the signal is distributed across a complex combination of many genes, and many or all of these external factors, we still need to acquire signatures salient to that given health or disease state in order to realize the vision of precision medicine. If a disease is truly complex, signatures and treatments are still required. A way to derive and then deconvolute these signatures – essentially – a high-resolution view of the *phenotype* is what we would need.

On the surface, having ways to account for all of these influences on their way to influencing a specific aspect of health or disease seems implausible but the chemistry of life may be a route to do just this.

### 1.3 Metabolomics – High-Resolution Phenotyping to Support Building The Blueprint?

The metabolome, in the framework for addressing the challenges cited above, can be defined as: *all the small molecules* (*metabolites*) *that circulate in the body.* These circulating metabolites (aka chemicals or biochemical) are from endogenous metabolic pathways, the microbiome, or from our environment – diet, chemical exposure, or supplements and drugs (prescription or elicit). Metabolomics is the technology for comprehensively surveying the entire metabolome from a single biological sample (akin to genomics for gene analysis). The use of metabolomics to survey the metabolome serves to inform about the health status or “phenotype” and all of its influences.

Many investigators have embraced metabolomics in the context of disease diagnosis and the response to drug treatment (i.e. pharmacometabolomics) and, in doing so, have made important contributions for defining discrete human phenotypes and associated response to treatments. The contributions are many and beyond the scope of this review, but the reader is referred to several contemporary reviews and examples [17], [18], [19], [20], [21], [22]. But, the more widespread use of metabolomics in precision medicine, particularly in the context of a cornerstone technology for precision medicine – genomics – is due to several important attributes of the metabolome. These attributes are described below.

**Fig. 2:**
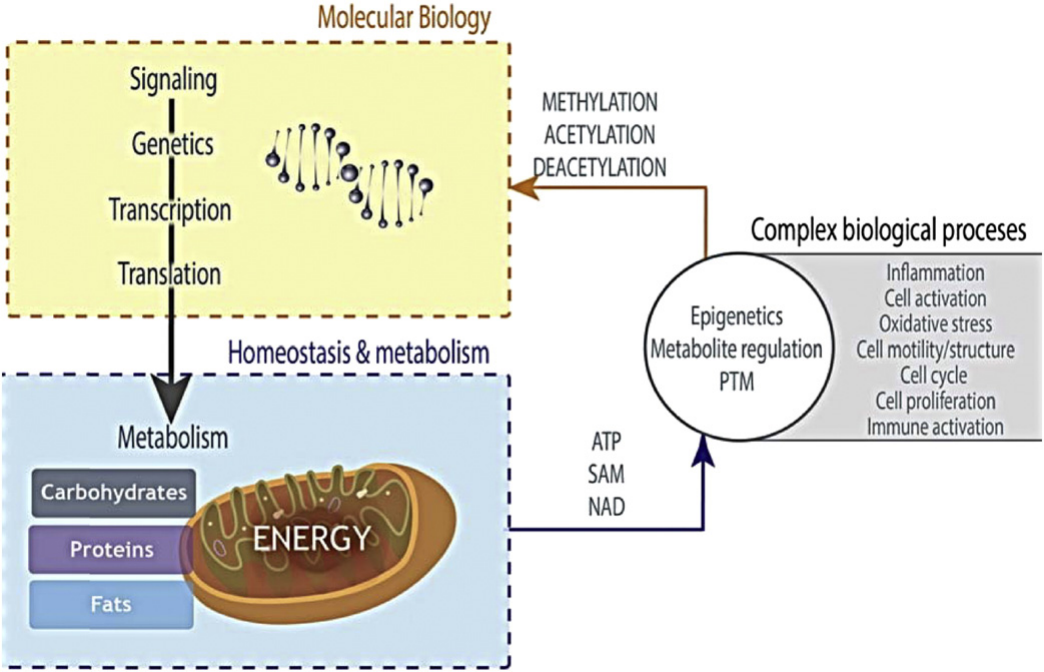
*Metabolic pathways are at the nexus of all cellular function*. Signaling through the central dogma of genes, transcripts, and proteins leads to metabolic pathway regulation. However, it is not always appreciated that metabolism also has a direct effect on posttranslational modifications, epigenetics, and consequently, on many complex biological processes (albeit, less well understood than the flow from genes to metabolic pathways).

The core of the metabolome maintains metabolic processes that represent the simplified unit that all life is organized around and strives to maintain homeostatically [19], [23], [24], [25]. Fig. 2 illustrates how metabolic pathways serve a central role in nearly all cellular processes – the source of energy, of DNA, proteins, cofactors, immune activation, motility, etc. – and how it is connected to molecular biological processes. The highly orchestrated process of metabolism required to support the most basic (e.g. energy production) and complex (e.g. cell division) cellular processes was established early in evolution and remains a focal point for all life [25], [26], [27]. Since metabolic processes are so tuned to cell/organism function, an individual's metabolic fingerprint reflects changes in homeostasis that underlie phenotypic changes [19], [28], [29]. And, as is increasingly appreciated, these biochemical pathways are intimately connected to nuclear function through metabolites such as ATP, SAM, Acetyl-CoA. All of these complex molecular biology networks are aimed at controlling physiological processes and metabolic pathways are at this nexus.

**Fig. 3:**
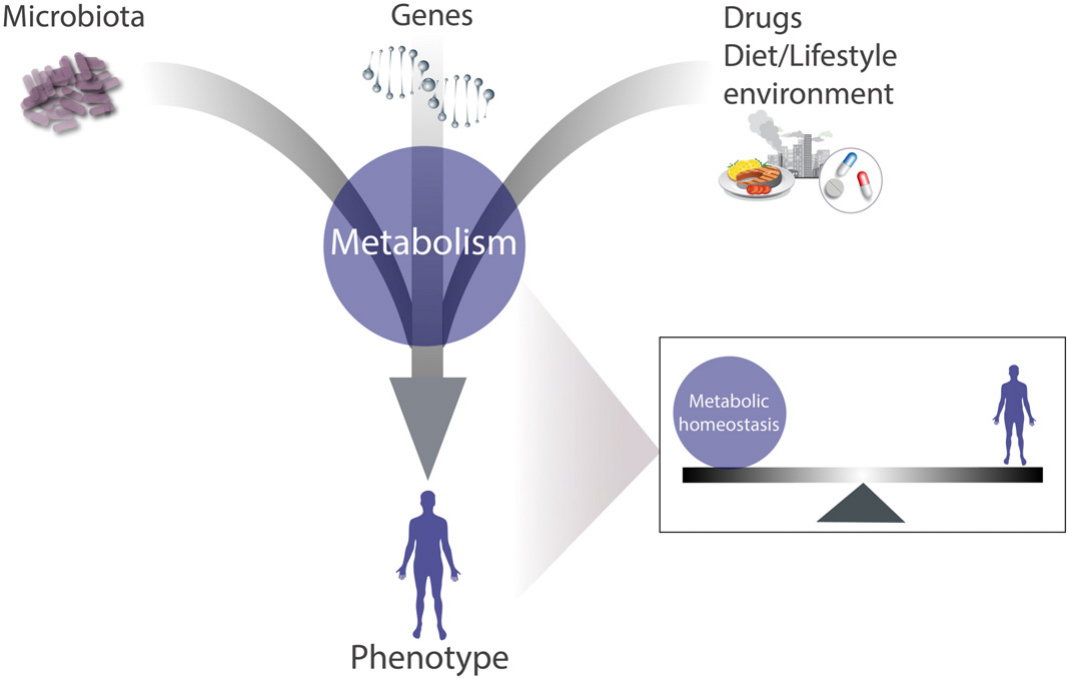
*Metabolism (metabolic pathways) are a surrogate of the phenotype*. Metabolites are ideally situated to account for phenotypic changes induced by either genes, the environment, the microbiota, or a complex combination of all of these influences.

Thus, metabolism is often regarded as being “diagnostic” of the phenotype itself [19], [30]. Metabolic homeostasis and the phenotype are coordinated, and hence, an alteration in one affects the other. Importantly, as we continue to uncover more about the complexity of the human phenotype and how most traits are combinatorially driven (i.e. genetics, microbiome, environment), metabolism offers a view of the functional state (Fig. 3) of an individual and can directly inform about the role of individual phenotypic drivers such as the following.

#### 1.3.1 Genetics

Genes alone frequently fail to explain the phenotype [31], [32]. But, whether it is a strong single gene variant (monogenic) or a complex set of alleles (polygenic) that induce phenotypic differences, if the phenotype is even subtly altered, metabolic changes almost invariably occur [1], [33]. Some particularly illustrative examples are where metabolic changes are shown to emerge even years before the onset of disease [34], [35], [36], [37] or an overt phenotype induced by a drug or toxicant treatment [38]. Many other examples could be cited but it is beyond the scope of this review to summarize all of the biomarker examples that illustrate the point that the effects of the genome (whether a monogenic or a polygenic trait) will frequently be recorded in an individual's metabolic profile.

#### 1.3.2 Environment, Diet, Lifestyle

Many traits are influenced by external factors. Examination of an individual's biochemical fingerprint in blood or urine may provide a signature for altered physiological processes that underlie phenotypic changes; furthermore, this signature can frequently be composed of clues about the absorption, distribution, metabolism, excretion (ADME) of dietary or environmental components that influence the phenotype in question. The genetic variation in any one of a myriad of steps involved in ADME, or the microbiome's variation, can influence the bioavailability of a metabolites. These differences in ADME can influence epigenetics [39], [40] (e.g. catechins in tea or curcumin in a curry dish) as well as the levels of essential cofactors and vitamins [41] (e.g. folate or choline). Thus, accounting for the levels of these biochemicals in the context of genetics, the microbiome and the health state will be important to deciphering traits.

#### 1.3.3 Microbiome

The last decade has revealed an additional layer of complexity to human health as more and more traits are shown to be substantially influenced by the microbiome [15] (such that some refer to it as a “second genome”). Microbial communities communicate using small molecules [42], so metabolomics is essential for deciphering this chemical output and the influence of this complex “organ” in the context of health and disease. Several traits such as autism, asthma, cancer, and obesity have been significantly associated with the composition of the microbiota and the examination of the metabolome using metabolomics has been an important element in deciphering function [43], [44], [45], [46], [47], [48], [49].

#### 1.3.4 Epigenetics

Lamarck's ideas on evolution, the development of traits as driven by environmental conditions, may have not have been as fallible as once thought. Studies continue to emerge showing that traits are not strictly inherited via DNA. Instead, some degree of inheritance (transgenerational) can be attributed to signals from the environment and transmitted epigenetically [26], [27], [50], [51], [52]. How widespread or relevant this is in mammals is yet to be concretely established. However, epigenetic or transgenerational inheritance is ultimately controlled by small molecule metabolites (e.g. SAM, Acetyl-CoA, etc) [20].

Numerous examples are cited that illustrate an intimate connection between metabolic status and epigenetics [26], [53]. And, new findings continue to emerge. For example, a plausible link between cellular metabolism, the epigenetic state, and stem cell fate was shown in the work by Shyh-Chang and colleagues where threonine levels were coupled to S-adenosylmethionine (SAM) regulation of histone methylation [54].

More extreme metabolic states like fasting or caloric restriction result in the production of the ketone body, D-b-hydroxybutyrate (BHBA). It was recently shown that D-b-hydroxybutyrate is an endogenous and specific inhibitor of class I histone deacetylases (HDACs). The resulting changes in histone acetylation and gene expression caused by BHBA promote stress resistance in the kidney [55].

As epigenetics continue to be deciphered in the context of genetics and health, it will be important to account for the small molecules (metabolites) that may in fact be the proximal signals for epigenetic activity.

#### 1.3.5 A Complex Combination of the Above

The connection of metabolites to the phenotype via the array of influences cited above offers a reminder that examining the chemistry of life (metabolites) has a rich heritage for building knowledge and making clinical decisions. In our current era of molecular biology, we often forget that some of the most fundamental aspects of physiology (cancer, muscle metabolism, diabetes, etc.) were mapped in detail by biochemists such as Cori, Warburg, Meyerhof, and Krebs. The result of their work in mapping discrete metabolic pathways was to uncover connections to higher order physiology – a coupling of physiology and metabolic pathways.

Urine chemical properties have guided physicians such as in establishing the concept of inborn errors in metabolism (IEMs) [29]. The idea suggested that a biochemical fingerprint within a biofluid (i.e. urine or blood) was a by-product of human variation and a surrogate for distinct pathologies. Garrod speculated that the inborn errors of metabolism that he was able to observe “were merely extreme examples of variations of chemical behavior which are probably everywhere present in minor degrees” [28]. In other words, he believed that there was a spectrum of phenotypes that could be associated with certain biochemicals. The technology available to him could not affirm this idea but it has been confirmed recently with metabolomics [19], [20].

More subtle phenotypes (e.g. susceptibility to a disease, response to a drug, prognosis) also produce a chemical fingerprint in biofluid [35], [38], [56], [57], [58], [59]. Hence, there is a strong and well-established association between the gene, the metabolite, and the biological state. Today, we utilize this knowledge as we assess our health by measuring metabolites such as glucose for diabetes and cholesterol for cardiovascular health. And, as discussed in the next section, biomarker discovery and validation efforts are producing a new pipeline of metabolite-based biomarkers that either are – or potentially will – produce the tests of tomorrow.

**Fig. 4:**
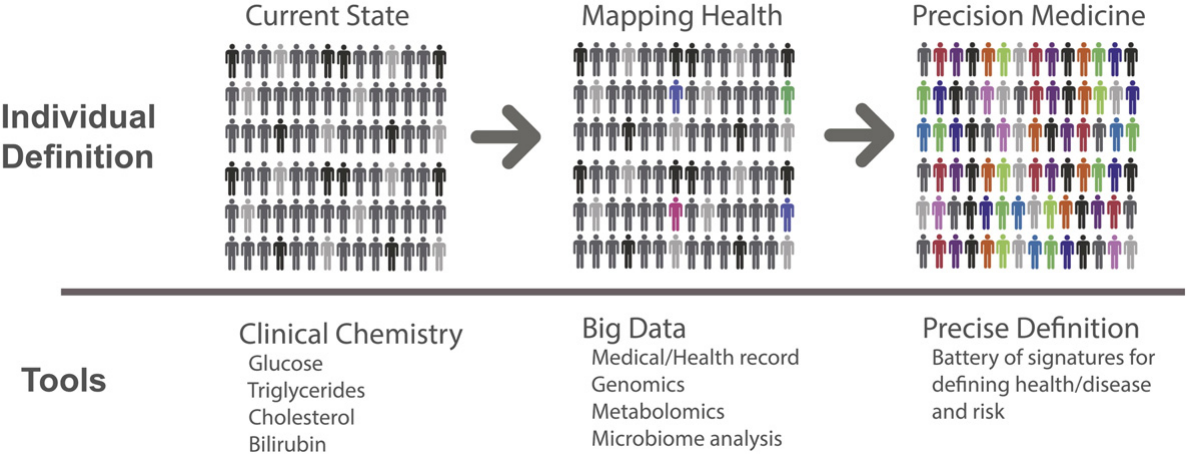
*Evolving landscape of precision medicine*. The majority of clinical tools today limit the ability to fully individualize a treatment for many conditions (left). Big data initiatives (middle) are currently enrolling and profiling thousands of individuals and collecting an immense amount of data at the clinical and molecular level to begin the process of defining individual health, disease, and response to interventions. As signatures (alleles, metabolite profiles) emerge (indicated by colored human silhouettes), they can be deployed as additional profiling and analysis reveals additional clinically useful signatures. The result of these initiatives aspires to offer the potential to define, track, and treat disease on a far more individual basis (far right).

Despite this heritage in elucidating our understanding of physiology by studying metabolic pathways and their routine clinical use, the last several decades have intensely focused on the genetic basis of disease and molecular biology. But, even a casual review of the literature shows that many researchers have not forgotten that metabolism is connected to all aspects of biology – from epigenetics – to metabolite signaling/regulation – to cell cycle and growth – to elaborate physiological processes like angiogenesis is demonstrating metabolism's key role in biology [25], [60], [61], [62]. Some assert that this return to metabolic inquiry is because the low-hanging fruit of molecular biology has been harvested, necessitating a deeper understanding of biological systems using this fundamental science [62]. With the maturity of metabolomics, an opportunity has arisen for precision medicine to ensure that this foundational metabolite data (in aggregate, the metabolome) can contribute to defining precision medicine signatures and mechanisms that can illuminate treatment strategy. Fig. 4 described how this is envisioned to evolve. Currently, precision medicine implementation options are relatively limited as treatments are based on the clinical assessment tools of the 20th century. Big data initiatives to characterize individual health, disease, and response with a battery of 'omic and clinical assessment tools are underway. Emerging from these initiatives will be a high-resolution view of health at the individual level, including additional signatures (biomarkers) of health and disease to support precision medicine at the highest level. From the vast number of data points provided through “omic discovery,” a reduced set of biomarkers will emerge into practice. In fact, biomarker signatures are already enriching precision medicine. As described below, metabolomics is continuing to play an important role in this evolution at several different levels – building the blueprint and deploying these signatures in a clinical setting as they emerge.

## 2 Case Examples

### 2.1 Building the Blueprint

**Fig. 5:**
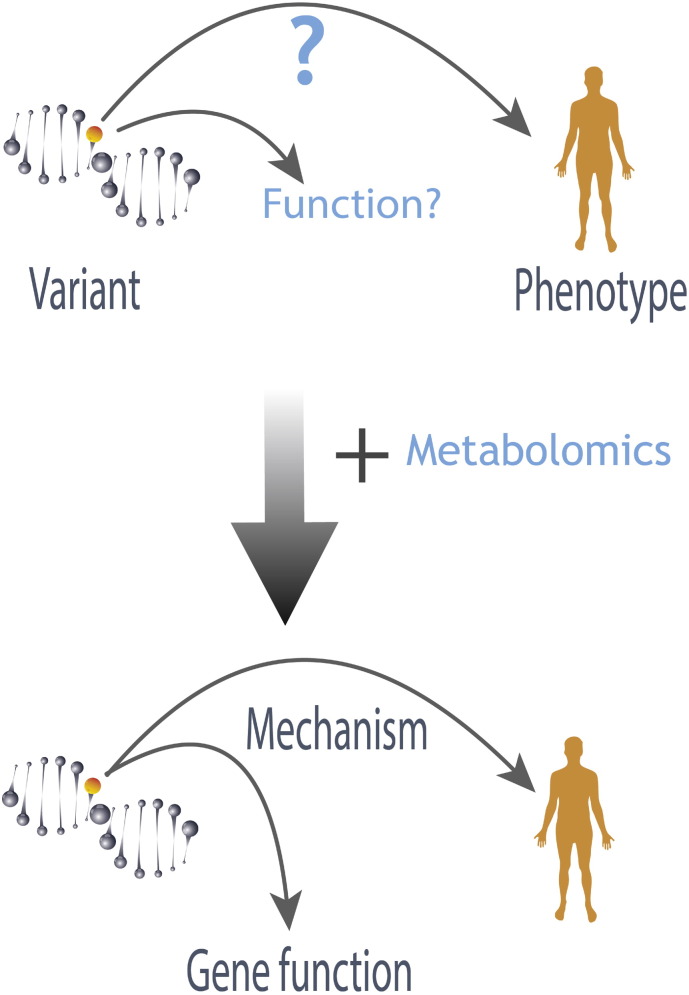
*Metabolomics is a genome “sentinel.”* Recent research 1, 2 showed that metabolites are a highly informative “intermediate phenotype” between genes and the phenotype. By illuminating alleles that actively perturb metabolism and reflecting back to the function of the allele, an awareness of metabolism's importance for understanding biology – particularly in the context of genetics – was re-kindled.

A cornerstone for realizing a more systematic and individualized version of precision medicine will be the collection of massive amounts of data to reveal signatures of various health and disease states and response to interventions. These efforts will involve the enrollment of large numbers of subjects and the collection of many data points on each subject through an array of methods including genomics, metabolomics, and microbiome profiling. This overall scenario is nicely reviewed elsewhere [7]. But it is important to point out that, as the data are being collected and analyzed, findings are emerging that can immediately provide clinical utility. As described below, metabolomics is a key element in these efforts as it effectively functions as a genome “sentinel” (Fig. 5).

For example, metabolomics on 2 large populations revealed an exceptionally strong association between the metabolite N-acetyl ornithine and the NAT8 locus [19], which was also reported to associate with kidney function. This information provided a dynamic marker for kidney function as N-acetylornithine concentrations were associated with kidney function (estimated glomerular filtration rate) and the risk allele of NAT8 was associated with higher N-acetylornithine concentrations. In another example of a very complex trait where genetic associations are elusive, interrogation of the metabolome revealed associations that provided insight about the etiology of the disease and provided dynamic biomarkers. This recent work described identifying a novel pathway of blood pressure regulation [63]. These are just 2 examples of how, as discoveries emerge from these large multi-omic population studies, they can enter a pipeline to further confirm and sharpen their clinical utility.

Thus, although thousands of individuals will be required to create a comprehensive map of individual health, the process is underway, and bearing useful signatures that can be deployed clinically today. Many of these associations were of known and suspected inborn errors of metabolism (IEMs) [20]. Fig. 4 illustrates how this is imagined to be evolving.

### 2.2 Disease Signatures (Biomarkers) in Action

As described above, a key utility of metabolomics is that it can complement data from other 'omics technologies. Specifically, the identification and quantitation of specific metabolites can be correlated to genetic sequence analysis which has been shown to identify associations with specific genotypes [19], [20].

One especially clear example of this is the use of metabolomics for inborn error testing (Fig. 5). Current diagnostics for detecting IEMs utilize kits which interrogate diagnostic biochemicals that increase or decrease in abundance based on the specific disease. Of the over 500 IEMs known to exist, 150–200 of these are more common and seen most often. These diseases result from mutations in genetic elements that affect metabolism. In order to test for these diseases, typically 10 or 20 individual clinical tests are needed which entails the testing of multiple sample types (e.g. whole blood, plasma, urine, cerebrospinal fluid). These tests interrogate specific enzyme activities or biochemically related groups of molecules (e.g., organic acids, amino acids). Thus, the current paradigm for diagnosing a patient is dependent on the selection of the right test or an exhaustive utilization of all the available test panels until a diagnosis is achieved. Further, diagnosis is dependent on the disease being canonical and therefore having an associated test clinically available.

**Fig. 6:**
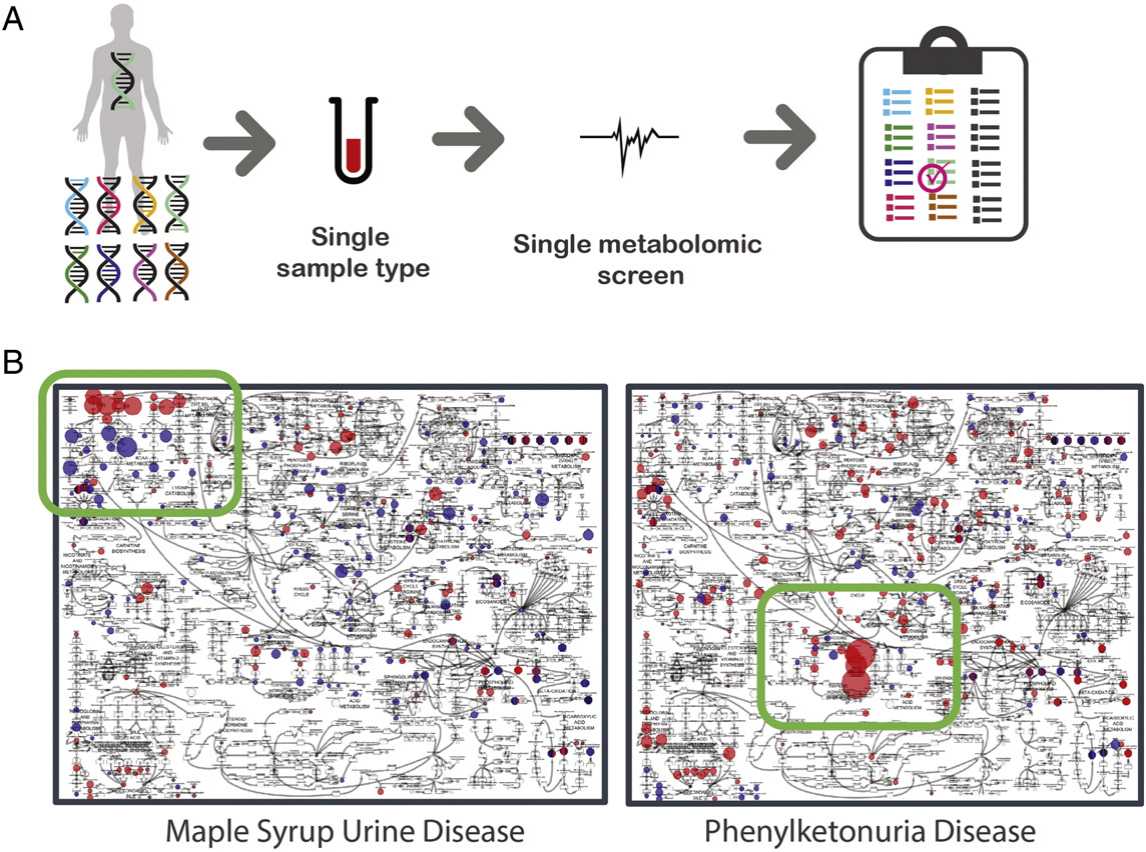
*Metabolomic screening to identify disease signatures – inborn errors of metabolism (IEMs)*. *A*. Paradigm for how a single sample submitted for a metabolomics screen can screen a multitude of genetic abnoramtilites at once. *B*. Two examples of patient samples overlaid on metabolic atlas where metabolites that are elevated or reduced relative to a control population are red and blue, respectively. The magnitude of the deviation is noted by the size of the colored sphere. In these examples, a clear pattern emerges from any normal metabolic variation and secondary disease effects to reveal the salient metabolic pathways and either maple syrup urine disease or phenylketonura. Adapted from Miller et al. [1].

A metabolomics workflow that can screen a single sample for thousands of biochemicals that comprise the canonical analytes in the test kits as well as additional molecules to aid in canonical and non-canonical disease would be a valuable approach for implementing precision medicine in the field of IEMs [64]. Indeed, Miller et al. recently described a promising start to this ideal [1]. Fig. 6 summarizes this work and how, from a single sample of plasma, signatures for nearly 20 IEMs could be identified [1].

Although metabolomics does not directly measure large molecules, this approach has also been utilized to screen for lysosomal storage disorders, congenital disorders of glycosylation, mucopolysaccharidoses, mucolipidoses, or other similar large molecule disorders as the small molecules that compose macromolecules can change in their respective pathways due to genetic defects.

Finally, this metabolomics approach may lead to the identification of new inborn errors in addition to also demonstrating that less invasive sample types can be screened to identify diseases that are currently diagnosed using test kits that require invasive sample types [33]. For example, nucleotide profiles are typically examined through testing of urine. However, the metabolomics workflow of plasma can identify nucleotides and nucleotide metabolites giving increased value to testing plasma if urine is not available or convenient for testing [1]. Additionally, the biomarkers of the neurological disorder aromatic amino acid decarboxylase deficiency can be identified in urine and plasma, when CSF is the front-line specimen of choice [65].

In addition to the identification of new biomarkers associated with specific genetic conditions such as those that cause inborn errors of metabolism, metabolomics will interrogate biomarkers associated with medicinal intervention, inflammation, disease status, and nutritional intervention, metabolomics also can identify xenobiotics, plant bi-products, and biochemicals linked to microbial metabolism. For example, recent work by Guo et al. combined metabolomics with whole exome sequencing (WES) of fairly healthy individuals revealing potentially damaging mutations that were not appreciated through WES data alone, and several metabolic indicators of early stage disease [2]. For example, one subject had profoundly elevated levels of fructose and the sugar alcohol sorbitol, but the patient had no complaint, and no damaging mutations within genes governing the metabolism of fructose or sorbitol were initially flagged. However, re-inspection of the WES data revealed a damaging mutation in the aldolase gene (ALDOB), suggesting a rationale for the elevated metabolites and providing a cautionary dietary planning note for this subject since fructose intolerance can result in organ damage. Although this was a small study (80 volunteers) of relatively healthy volunteers, the information derived from metabolomics suggested that, even in more subtle health states, metabolomics alleviates some of the challenges of genomics by identifying genes that are effectively “active” and then helping to create a functional connection between the gene and the health state.

## 3 Conclusions

Precision medicine is a medical model that customizes health care decisions, medical practices, and products to the individual. Implementation of specific diagnostic testing and therapeutic intervention can be selected based on the context of a patient's biological signatures (i.e. genetic, biochemical, or otherwise). This tailored approach to diagnosis and treatment has the potential to realize greater efficacy while limiting costs and side effects. Clearly, there is more work to do in order to expand the repertoire of precision medicine options to more individuals and disease states but useful signatures (biomarkers) are emerging.

With the availability of 'omic technologies, researchers and clinicians can now generate large amounts of data on biological samples. The maturation of bioinformatics technologies is now allowing for the analysis of large data sets such as entire genomes in shorter time frames. The ability to practice precision medicine does and will continue to depend on the knowledge acquired through the analysis of cohorts of clinical samples. Tools or interfaces that increase the utility of these 'omics in routine practice will help to drive the production and availability of the knowledge base in precision medicine to ultimately assist clinicians in taking action based on the results. Improvements to data acquisition, data analysis, and data utilization will drive precision medicine initiatives such as those proposed by President Barack Obama in his 2015 State of the Union address.

And, while genomics is a mainstay for deriving a higher resolution view of human health – the view that will yield the roadmap for tailored individual healthcare – metabolomics is a clear ally. Box 1 highlights the major roles of metabolomics in current and emergent precision medicine – from large cohort analysis to individual health and risk assessment.

A main focal point for where metabolomics fits into this is its relationship to the phenotype whether the phenotype is primarily driven by a single gene or a complex combination of external factors. Associating biochemical levels and alterations with specific genotypes or external factors such as the microbiome offers the ability to streamline diagnostics and utilize a greater breadth of information to the clinic to assess patient health.

## Box 1 The role of metabolomics in precision medicine.

A framework for understanding metabolomics' role in precision medicine can be summarized under the following 4 points. Metabolomics has the direct ability to:

1. *Phenotype individuals*
  • Even in cases where important traits yield no (or very weak) associations to genetics [57], [59], [63]
  • Or in cases where the phenotype is driven by environmental, microbiota, or epigenetics [49]
  • Or undifferentiated phenotypes (e.g. child with developmental delay, seizures, autism, etc.) [66]
  • Metabolic pathways associating with a phenotype may contribute to mechanistic understanding of the phenotype.
2. *Identify alleles and mutations with biological significance – penetrant alleles*
  • Alleles that actively change metabolism are disproportionally important phenotypically [20]
  • Metabolites act as a “sentinel” to active genes to assist in avoiding false discoveries or missing important alleles that, by themselves, have a small effect size [19], [20]
  • With equivocal molecular test in a gene known to be involved in metabolism
3. *Identify allele function*
  • Through the direct functional association of metabolites and genes [19], [20]
  • Even in the “dark matter,” non-coding regions (introns) [67], [68]
  • Variants of unknown significance (VUS) [2]
4. *Perform longitudinal analysis of individuals by following dynamic metabolite markers*
  • Track treatment through the interrogation of metabolites of the treatment regimen
  • Track disease progression or response to treatment with dynamic metabolite markers
